# Monoclonal Antibodies against Hepatitis C Genotype 3a Virus Like Particle Inhibit Virus Entry in Cell Culture System

**DOI:** 10.1371/journal.pone.0053619

**Published:** 2013-01-15

**Authors:** Soma Das, Rohini K. Shetty, Anuj Kumar, Radhika Nagamangalam Shridharan, Ranjitha Tatineni, Giriprakash Chi, Anirban Mukherjee, Saumitra Das, Shaila Melkote Subbarao, Anjali Anoop Karande

**Affiliations:** 1 Department of Biochemistry, Indian Institute of Science, Bangalore, India; 2 Department of Microbiology and Cell biology, Indian Institute of Science, Bangalore, India; University of Hyderabad, India

## Abstract

The envelope protein (E1–E2) of Hepatitis C virus (HCV) is a major component of the viral structure. The glycosylated envelope protein is considered to be important for initiation of infection by binding to cellular receptor(s) and also known as one of the major antigenic targets to host immune response. The present study was aimed at identifying mouse monoclonal antibodies which inhibit binding of virus like particles of HCV to target cells. The first step in this direction was to generate recombinant HCV-like particles (HCV-LPs) specific for genotypes 3a of HCV (prevalent in India) using the genes encoding core, E1 and E2 envelop proteins in a baculovirus expression system. The purified HCV-LPs were characterized by ELISA and electron microscopy and were used to generate monoclonal antibodies (mAbs) in mice. Two monoclonal antibodies (E8G9 and H1H10) specific for the E2 region of envelope protein of HCV genotype 3a, were found to reduce the virus binding to Huh7 cells. However, the mAbs generated against HCV genotype 1b (D2H3, G2C7, E1B11) were not so effective. More importantly, mAb E8G9 showed significant inhibition of the virus entry in HCV JFH1 cell culture system. Finally, the epitopic regions on E2 protein which bind to the mAbs have also been identified. Results suggest a new therapeutic strategy and provide the proof of concept that mAb against HCV-LP could be effective in preventing virus entry into liver cells to block HCV replication.

## Introduction

Hepatitis C virus (HCV) is the major etiological agent of non-A, non-B hepatitis that infects almost 200 million people worldwide [1]. HCV is a major cause of post transfusion and community-acquired hepatitis. Approximately 70–80% of HCV patients develop chronic hepatitis of which 20–30% leads to liver disease, cirrhosis and hepatocellular carcinoma [2]. Treatment options for chronic HCV infection are limited, and a vaccine to prevent HCV infection is not available.

The virion contains a positive-sense single stranded RNA genome of approximately 9.6 kb that consists of a highly conserved 5′ non coding region followed by a long open reading frame of 9,030 to 9,099 nucleotides (nts). It is translated into a single polyprotein of 3,010 to 3030 amino acids [3], [4]. A combination of host and viral proteases are involved in the polyprotein processing to generate ten different proteins. The structural proteins of HCV are comprised of the core protein (∼21 kDa) and two envelope glycoproteins E1 (∼31 kDa) and E2 (∼70 kDa) [3]–[5]. E1 and E2 are transmembrane proteins consisting of a large N-terminal ectodomain and a C-terminal hydrophobic anchor. E1 and E2 undergo post translational modifications by extensive N-linked glycosylation and are responsible for cell binding and entry [6]–[15].

Due to the error-prone nature of HCV RNA-dependent RNA polymerase and its high replicative rate *in vivo*, it shows a high degree of genetic variability [8]–[9]. Based on the sequence heterogeneity of the genome, HCV is classified into six major genotypes and ∼100 subtypes. These six genotypes of HCV differ in their pathogenicity, efficiency of translation/replication and responsiveness to antiviral therapy. Genotypes 1 and 2 are the major types observed in Japan, Europe, North America and South-East Asia respectively. Type 4 has been found in Central Africa, Middle East and Egypt, type 5 is found in South Africa and type 6 in South-East Asia [16]. Interestingly, the entire gene sequence of HCV genome shows >30% divergence at the nucleotide level across all the genotypes [16]. Unlike in the other parts of the world, genotype 3 has been found to be predominant in India and infects 1% of the total population, followed by genotype 1 [17].

Although a detailed analysis of the viral genomic organization has led to the identification of various genetic elements and the establishment of subgenomic replicons, the study of viral attachment and entry is still not studied completely due to the inability of the virus to propagate efficiently in cell culture and the lack of suitable animal model for the virus.

Several groups have described the generation of HCV-like particles (HCV-LPs) in insect cells using a recombinant baculovirus containing the cDNA of the HCV structural proteins core, E1 and E2 [18]–[23]. In contrast to individually expressed envelope glycoproteins, the HCV structural proteins have been shown to assemble into enveloped HCV-LPs with morphological, biophysical, and antigenic properties similar to those of putative virions isolated from HCV-infected humans [18]–[19], [24]–[25] . They may, therefore, interact with anti-HCV antibodies directed against HCV envelope proteins that may represent neutralizing epitopes. Recent studies have demonstrated that HCV-LPs interact with defined human cell lines and hepatocytes similar to viral particles isolated from human serum. The interaction of HCV-LPs with permissive cell lines therefore represents a novel model system for the study of viral binding and entry and consecutively inhibition of entry into permissive cells [21], [23], [25]–[27].

In the present study, we have generated HCV-LPs comprising of core-E1-E2 regions of genotypes 1b and 3a using the baculovirus expression system and these HCV-LPs have been used to produce mouse monoclonal antibodies. These monoclonal antibodies were characterized for their ability to inhibit VLP attachment to human hepatoma cells and also virus entry into Huh 7.5 cells in infectious cell culture system.

## Materials and Methods

### Ethics Statement

The animal experiments have been approved by the 'Institutional Animal Ethics Committee', Indian Institute of Science, Bangalore, India. Mice were housed in 12 hr night-day cycle at controlled temperature of 24 degree centigrade and humidity and food *ad libitum*.

### Cell Culture

Huh 7 and Huh7.5 cells [28] were maintained in Dulbecco’s modified Eagle medium (DMEM, Sigma) supplemented with 10% fetal bovine serum at 37°C under 5% CO_2._ Sf21 cells were maintained in TC100 insect cell Medium (Sigma) with 10% fetal bovine serum at 26°C.

### Generation of HCV-LPs

The sequence encoding core-E1-E2 for genotype 3a from cDNA corresponding to RNA isolated from patient blood has been cloned in pGEMT Easy vector (Acc. No. core: GU172376 and E1E2: GU172375). The core-E1-E2 region was subsequently subcloned in pFastBac HTb at BamHI-EcoRI site (2.256 kb). Similarly, the core-E1-E2 of genotype 1b was amplified from replicon Con 1FL (Acc. No. AJ238799) [29] and cloned into pFastBac HTc in frame. After the generation of bacmid, integration of DNA specific for core-E1-E2 into the baculoviral genome was confirmed by PCR amplification using M13F and E2R primers for genotype 3a or core F and M13R primers for genotype 1b. The recombinant baculoviruses were rescued from the bacmid and the viruses were amplified in Sf 21 cells. Time course expression of the core-E1-E2 protein in insect cells by recombinant baculovirus was tested 24, 48, 56 and 72 h of post infection at 10 moi. Wild type baculovirus infection cell extracts were used as controls.

### Purification of HCV-LPs

Sf21 cells were infected with recombinant baculovirus at a moi of 5–10, and cells were harvested 72 h post infection. Cell pellets were washed with phosphate buffered saline (PBS: 50 mM phosphate buffer pH 7.2 containing 150 mM NaCl) thrice and were resuspended using a tissue homogenizer in a lysis buffer (50 mM Tris, 50 mM NaCl, 0.5 mM EDTA, 1 mM PMSF, 0.1% NP40 and 0.25% protease inhibitors). The lysate was centrifuged at 1500×g for 15 min at 4°C and the supernatant was pelleted over a 30% sucrose cushion. The pellet was resuspended in 20 mM Tris and 150 mM NaCl which was then applied on a 20% to 60% sucrose gradient in SW41 rotor (Beckman). After 22 h of ultracentrifugation at 30,000 rpm at 4°C, fractions (1 ml) were collected and tested for E1 and E2 by enzyme-linked immunosorbent assay (ELISA) and western blotting. Anti E1–E2 polyclonal antibody raised in rabbit was used for the above assays. Fractions containing HCV-LPs were diluted with 10 mM PBS and pelleted at 30,000 rpm for 2 h and stored at −70°C. Protein concentration was determined by Bradford protein assay reagent.

### Electron Microscopy of HCV-LPs

Purified HCV-LP samples (5 µl of 2 µg/ml concentration) were absorbed on the surface of carbon coated 300 mesh copper grids for 1 min, and negatively stained with 2% uranyl acetate and observed under a transmission electron microscope (Tecnai F30 FEI-Eindhoven, Netherlands) at magnification of 10,000X and 20,000X.

### Analysis of Binding of HCV-LPs to Huh7 Cell Lines

To analyse the binding of HCV-LPs to Huh7 cells, 5×10^5^ cells were incubated with HCV-LPs of different concentrations in PBS (final volume-100 µl) for different time points at 37°C. Unbound HCV-LPs were removed by washing with 0.5% BSA in PBS. Cells were subsequently incubated for 1 h at room temperature with anti-E1E2 polyclonal antibody followed by incubation with FITC-conjugated anti-rabbit IgG antibody. Cell-bound fluorescence was analyzed using FACS Calibur flow cytometer (Becton Dickinson) using WinMDI software to calculate the mean fluorescence intensity (MFI) of the cell population, which directly relates to the surface density of FITC-labelled HCV-LPs bound to hepatocytes [30]. The MFI values of cells with or without HCV-LPs and with isotype control antibody were compared. OVCAR 3 cells (ovarian carcinoma) were used as negative control cells for binding of VLP (data not shown).

### Immunization of Mice and Establishment of Hybridoma

Purified VLP (30 µg for each mouse) emulsified with Freund’s adjuvant was administered subcutaneously to 6–8 weeks old female BALB/c mice three boosters (15 µg for each mouse) at interval of three weeks. After a month, the mice were finally injected intraperitoneally with 100 µg of the antigen in saline and four days later the animals were sacrificed. The spleens were excised, and the splenocytes were fused with Sp2/0 mouse myeloma cells using polyethylene glycol 4000 (Merck). Hybridoma were selected on HAT (Hypoxanthine-aminopterin-thymidine medium) supplemented by IMDM subsequently. Hybridoma obtained were tested for specific antibody production using ELISA and subcloned to obtain single cells. Monoclonal antibodies (mAbs) were purified from culture supernatant by affinity chromatography on a protein A-Sepharose column by following standard procedures [31].

### Immunoassays. (i) ELISA

Microtiter ELISA plates (Nunc) were coated overnight with antigen (HCV-LP) (5 µg/ml in PBS) followed by blocking of unoccupied sites with 0.5% gelatin in PBS. The plates were incubated with different culture supernatant samples. After three washes with PBS containing 0.05% Tween 20, the plates were incubated with rabbit anti-mouse Ig-HRP conjugate (DAKO, Glostrup, Denmark) for 1 h. The bound-peroxidase activity was detected using tetramethylbenzidine (TMB) and 0.03% H_2_O_2_. The reaction was stopped with 1 M H_2_SO_4_, and absorption at 450 nm was measured in an ELISA plate reader (Spectramax; Molecular Devices).

### (ii) Western Blotting

HCV-LPs were electrophoresed on 10% polyacrylamide gel under reducing conditions and transferred onto nitrocellulose membranes. After blocking the non-specific sites with 0.5% BSA in PBS, the membranes were incubated with mouse antibodies specific to the HCV-LP, followed by rabbit anti-mouse Ig-HRP conjugate. The blot was developed with diaminobenzidine (1 mg/ml in citrate buffer, pH 5.0, containing 0.05% H_2_O_2_) or Enhanced Chemiluminescence.

### Inhibition of Binding of HCV-LP to Huh7 Cells by Monoclonal Antibodies against Genotypes 1b and 3a

HCV-LPs were pre-incubated with different concentrations of purified individual monoclonal antibodies and the complexes were allowed to react with Huh 7 cells. The binding of the labeled VLPs was monitored by flow cytometry analysis as described above.

### Identification of the Epitopic Regions Recognized by the mAbs

A set of five overlapping E2 gene fragments were generated by PCR amplification followed by restriction enzymes (Bam HI and Hind III) digestion of the different regions of E2 gene and subcloned. The corresponding protein fragments were expressed in *E. coli.,* purified and used for western blot analysis. The fragments R1 (16.94 kDa), R2 (10.78 kDa) R4 (11.44 kDa) and R5 (11.11 kDa) were cloned in pRSET B vector, whereas R3 (12.65 kDa) was cloned in pRSET A vector. In the fragment R3, a part of the vector sequences (∼2.5 kDa) was included in the expressed protein, however that part did not contribute to the reactivity to the mAb E8G9 (data not shown).

### *In vitro* Transcription of Viral RNA

The pJFH1 construct (generous gift from Dr. Takaji Wakita, National Institute of Infectious Diseases, Tokyo, Japan) was linearized with XbaI. HCV RNA was synthesized from linearized pJFH1 template using Ribomax Large scale RNA production system-T7 according to manufacturer’s instructions (Promega).

### Transfection and Generation of JFH1 Virus

Huh7.5 cells were transfected with *in* *vitro* synthesized JFH1 RNA transcript using Lipofectamine 2000 (Invitrogen) in Opti-MEM (Invitrogen). Infectious JFH1 virus particles were generated as described previously [28]. Uninfected Huh7.5 cells were used as a mock control.

### Virus Neutralization Assay

Anti-E2 antibodies (E8G9 and H1H10) generated against genotype 3a VLP were tested for their ability to neutralize virus infectivity. Huh7.5 cells were seeded into 24 well plate 16 h prior to the day of infection. JFH1virus was incubated with serial dilutions of E2 mAbs at 37°C for 1 hr. The antibody-virus mixture was then transferred on the cells. Infectivity was analyzed three days (for HCV negative sense strand detection) or three hours (for input HCV positive sense strand detection) post infection by real-time RT- PCR.

### Quantification of Viral RNA

Viral RNA was quantified by real-time RT-PCR analysis. Cells were harvested three hours (for HCV positive sense strand detection) or three days (for HCV negative sense strand detection) post infection and total RNA was isolated which was reverse transcribed with HCV 3′ primer (for positive sense) or HCV 5′ primer (for HCV negative sense) and GAPDH 3′primer using Revert-Aid (Thermo Scientific). Resulting cDNA was amplified for HCV IRES and GAPDH (internal control) using the ABI real time RT-PCR System (Applied Biosystems).

## Results

### Characterization of HCV-LPs of Genotypes 1b and 3a

**Figure 1:**
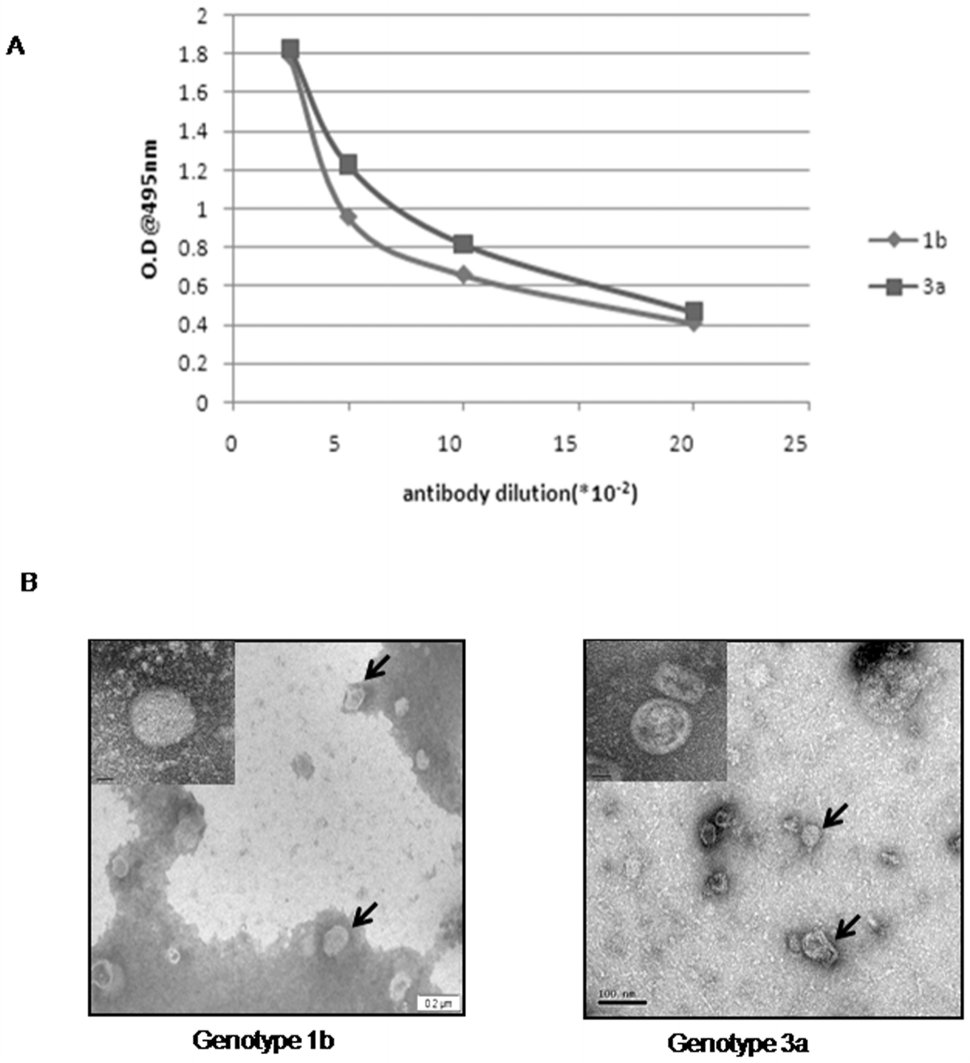
Characterization of HCV-LPs. (A) HCV-LPs corresponding to genotypes 3a and 1b were harvested on 4^th^ day post infection and purified as described in Materials and Methods. HCV-LPs were tested with different concentrations of anti-HCV-E1E2 antibody using ELISA. (B) Transmission electron microscopy of HCV-LPs of 1b and 3a as indicated. Scale bar: 200 nm for genotype 1b and 100 nm of genotype 3a; magnification: 10,000X. (Inset shows a single virus particle with 20,000× magnification).

HCV-LPs corresponding to genotypes 1b and 3a (comprising of core-E1–E2) have been generated using the baculovirus expression system in insect cells. The purified HCV-LPs of both genotypes were tested for immunoreactivity with polyclonal antibody to recombinant E1–E2 (Fig. 1A). The particles were further examined under electron microscope (Fig. 1B). Results showed particles of 40–60 nm size of genotype 1b which was similar to the sizes described earlier [18] and 35–55 nm for genotype 3a. The size difference may be due to the difference in the amount of E1 and E2 proteins incorporated into each virus like particle.

The purified HCV-LPs binding to Huh7 cells were analyzed by flow cytometry at 37°C. It was observed that with constant concentration of VLP (7 µg), at different time points, the intensity of fluorescence increased gradually upto 4 h which declined afterwards (Figure S1).

Further, the binding efficiency of the HCV-LPs was compared at 4^th^ hr time point. HCV-LP corresponding to genotype 3a showed marginally higher interaction (∼80%) with the Huh7 cells than the HCV-LP of genotype 1b (∼70%) (Figure S2).

### Characterization of Monoclonal Antibodies Against 1b and 3a Genotype of HCV-LP

BALB/c mice were immunized with the HCV-LPs (both genotype 1 and genotype 3) and hybridoma were established by fusion of splenocytes with mouse myeloma cells. Approximately 200 hybridomas from two independent experiments were screened. A total of five mAbs were obtained out of which two (E8G9 and H1H10) were against genotype 3a and three (E1B11, D2H3 and G2C7) were against genotype 1b. The cross reactivity of the monoclonal antibodies was determined by ELISA employing HCV-LP of other genotype as coating antigen (500 ng). As seen in Table 1, mAbs E8G9 against 3a HCV-LP and G2C7 against 1b HCV-LP showed maximum reactivity and were also cross reactive with both HCV-LPs to the same extent. mAbs E8G9 and D2H3 reacted strongly with the envelope protein in Western blot analysis suggesting that they recognize linear epitopes. The other three mAbs (E1B11, G2C7 and H1H10) reacted well in ELISA and dot blot but not in Western blot indicating that they are generated against conformational epitopes. The characteristics of the monoclonal antibodies are summarized in Table 1.

**Table 1:** Reactivities and epitope mapping of monoclonal antibodies (mAbs) against HCV-LPs of genotypes 3a and 1b.

mAb	Epitopic region on E2	WB	ELISA/Dotblot	Titer (HCV-LP 3a)	Titer (HCV-LP 1b)
G2C7	ND	−	++	Beyond 1024	Beyond 1024
E8G9	aa 555–699	++	++	Between 256 and 512	256
H1H10	ND	−	+	Between 8 and 16	Between 8 and 16
D2H3	aa 596–699	+	+	Between 64 and 128	Between 128 and 256
E1B11	ND	−	+	64	64

### Inhibition of HCV-LP Binding to Huh 7 Cells by mAbs

**Figure 2:**
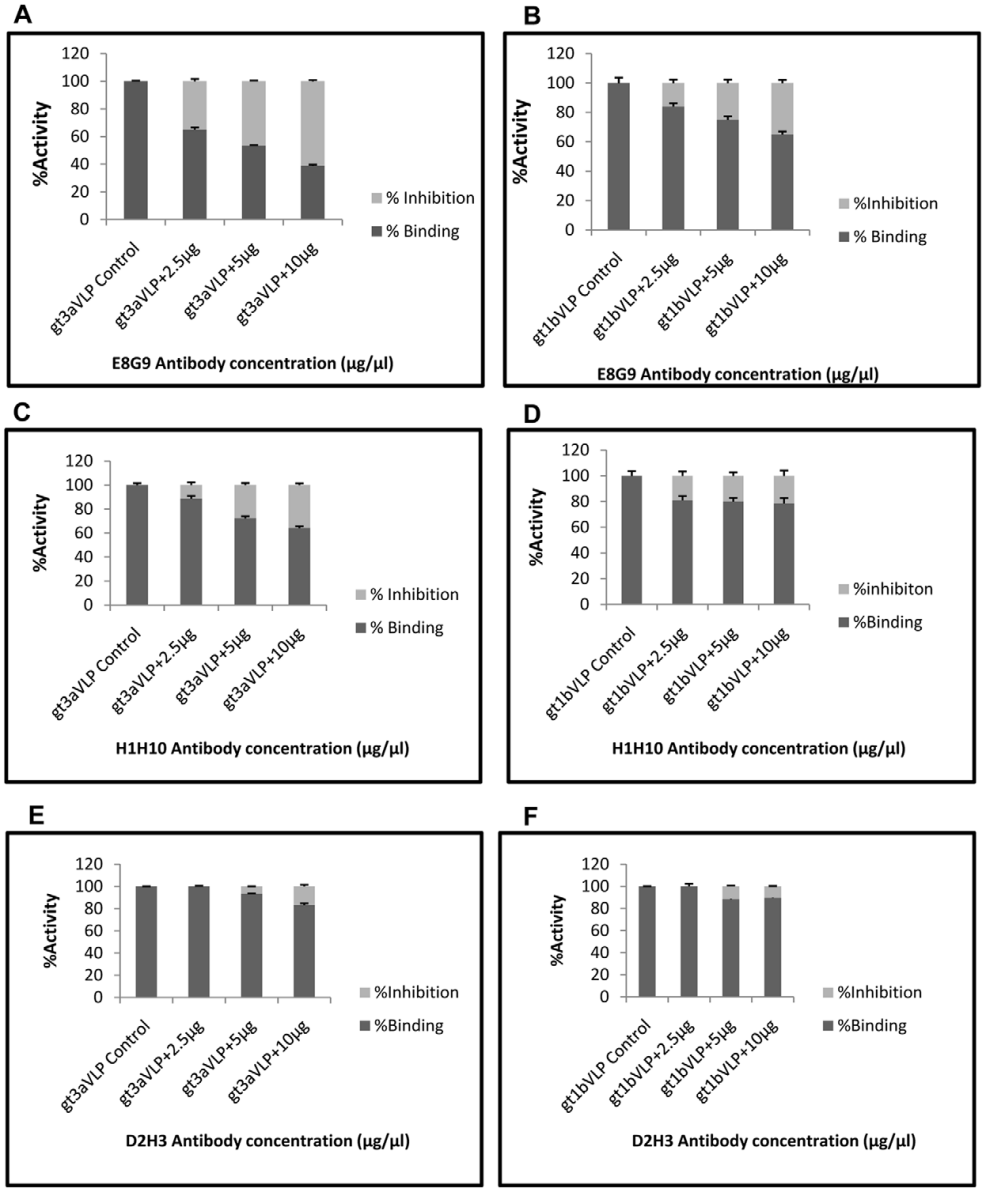
Inhibition of HCV-LP and Huh7 cell binding by mAbs. HCV-LPs of both genotypes 1b and 3a were incubated with increasing concentrations of mAbs as indicated. The Y-axis depicts the percentage activity representing both the percent binding (dark grey) and percent inhibition HCV-LP attachment (light grey).

Since all the mAbs exhibited cross-genotype specificity in reactivity, it is likely that these mAbs would inhibit binding of HCV-LPs to Huh7 cells. To explore this possibility, increasing concentrations of mAbs were incubated with constant amount HCV-LPs and the binding of HCV-LPs to Huh 7 cells was monitored. The inhibition of HCV-LPs binding by mAbs was determined by flow cytometric analysis. Results showed dose dependent inhibition of binding of the HCV-LP with increasing concentrations of the mAb E8G9 (∼66%). Considerable inhibition was also observed with mAb H1H10 (∼30%). However all other mAbs did not show appreciable inhibition of the binding (Fig. 2). The results are tabulated in Table 2 and Table 3. A non-specific antibody F1G4 has been used as a negative control [32] (Figure S3).

**Table 2:** Percentage inhibition of HCV-LP genotype 3a binding to Huh 7 cells using monoclonal antibodies.

	Percentage inhibition of binding		
mAb	10 µg	5 µg	2.5 µg
G2C7	1	0	0
E8G9	66	45	26
H1H10	30	26	12
D2H3	3	6	0
E1B11	0	0	0
Non-specific	0	0	0

**Table 3:** Percentage inhibition of HCV-LP genotype 1b binding to Huh 7 cells using monoclonal antibodies.

	Percentage inhibition of binding		
mAb	10 µg	5 µg	2.5 µg
G2C7	0	0	0
E8G9	25	24	14
H1H10	29	23	23
D2H3	12	2	2
E1B11	0	0	0
Non-specific	0	0	0

### Inhibition of Virus Entry by the mAbs in HCV Cell Culture

**Figure 3:**
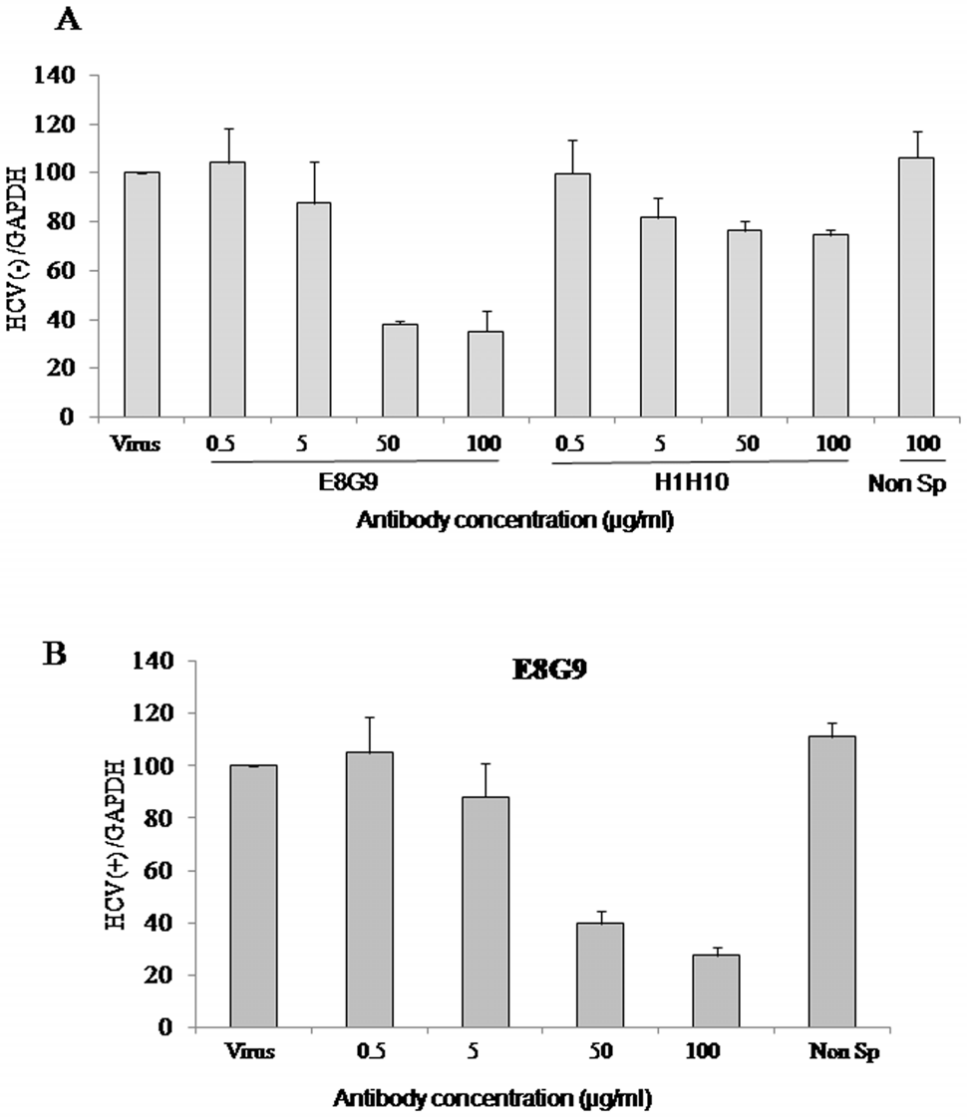
Effect of mAbs on HCV infection. (**A**) JFH1 virus was preincubated with increasing concentrations (0.5, 5, 50, 100 µg/ml) of mAbs (E8G9 and H1H10) or 100 µg/ml of non-specific antibody F1G4 (Non sp) for 1 hr at 37°C before infecting Huh7.5 cells. Three days post infection, total cellular RNA was isolated and HCV negative strand level was measured using real time RT-PCR. GAPDH was used as an internal control. (**B**) mAb E8G9 was used in increasing concentrations to inhibit the virus entry and the input viral RNA present inside the cells (positive strand) was estimated 3 h post infection by real time RT-PCR. GAPDH was used as an internal control.

Flow cytometric analysis suggested that mAbs E8G9 and H1H10 were able to inhibit the HCV LPs binding to Huh7 cells. To verify whether this property is also shown when virions of hepatitis C are used, neutralization assays were performed using JFH1 virus. The virus was pre-incubated with different concentrations of the antibodies (EG89 & H1H10) specific for HCV-LP (genotype 3a) for 1hr at 37°C before infection. An unrelated monoclonal antibody (F1G4) was used as negative control. Three days post infection, the effect of antibodies on HCV negative strand synthesis was measured by real time RT-PCR. Huh7.5 cells infected with JFH1 virus in the presence of 100 µg/ml E8G9 mAb showed nearly 65% reduction in intracellular HCV RNA level, while H1H10 showed a modest decrease of about 20% at the same concentration and non specific antibody did not show any inhibition (Fig. 3A).

To further confirm that this inhibition of HCV negative strand synthesis by E8G9 antibody is due to inhibition of virus entry, we performed *in vitro* neutralization assay and quantified the level of input positive strand three hours post infection using real time RT-PCR (Fig. 3B). A significant reduction in virus entry at 50 and 100 µg/ml was observed with E8G9 mAb suggesting it as a good candidate for inhibiting HCV entry in cell culture system.

### Epitope Mapping of mAbs

**Figure 4:**
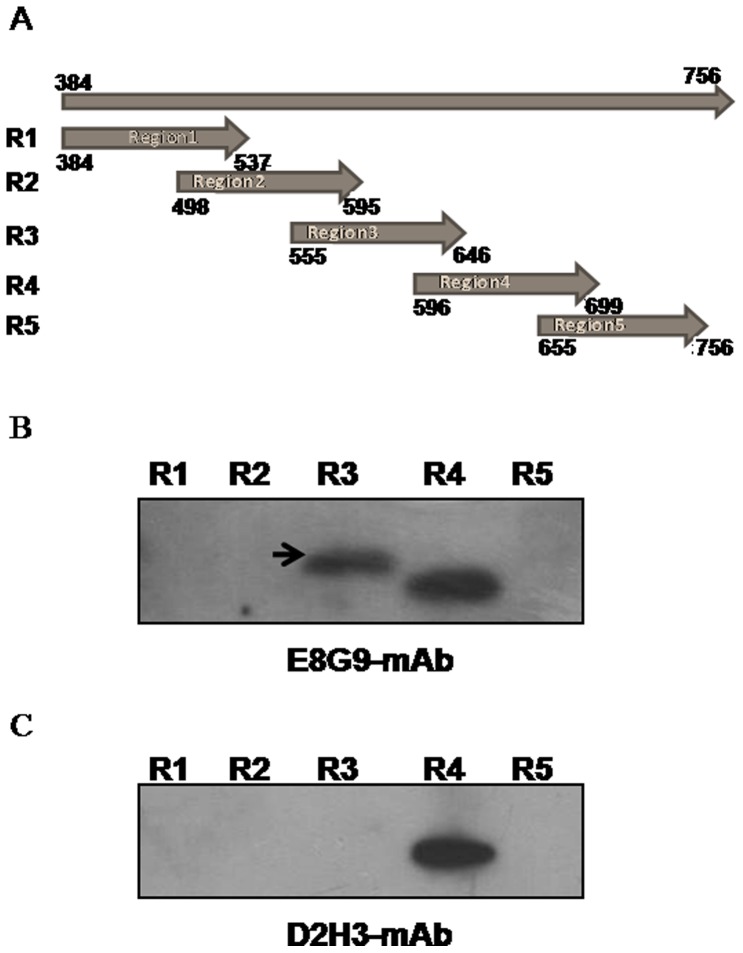
Epitope mapping of E8G9. (**A**) Schematic representation of different fragments of HCV E2 protein used for epitope mapping. (**B**) Western blot analysis of the recombinant proteins from five regions of E2 (region 3 specific for E8G9 is indicated using an arrow). (R1–R5 denote different regions).

The inhibition of binding of HCV-LPs to Huh 7 cells by E8G9 and not by D2H3 may be due to non-overlapping epitopic regions recognized by the two mAbs, E8G9 and D2H3. To delineate the specific epitopic regions, western blot analysis was carried out using different overlapping fragments of HCV E2 protein (Fig. 4A), expressed in *E. coli*. The entire E2 coding region of HCV was divided into five overlapping gene fragments (Fig. 4A), which were amplified, cloned and expressed in *E. coli*. All the five purified protein fragments were analyzed by western blot analysis with E8G9 and D2H3 mAbs. It was seen that E8G9 reacted with region 3 (555 to 646 aa) and region 4 (596 to 699 aa) whereas mAb D2H3 reacted with region 4 only (Fig. 4B). Results indicated that region 3 which is present between amino acids 555 to 646 may be involved in the inhibition of HCV-LP binding to Huh 7 cells. The epitope of mAb H1H10, could not be delineated because it recognizes a conformational epitope and thus fails to react in western blot analysis.

## Discussion

In this work, we have reported for the first time the generation of recombinant HCV-LP for genotype 3a, which is prevalent in India. We have also generated the HCV-LP corresponding to genotype 1b prevalent worldwide for comparison. The HCV-LP corresponding to 1b appears to be polygonal in shape and 40 to 60 nm in size as reported earlier, whereas HCV-LP of 3a was found to be approximately 35–55 nm in size. Thus, structurally and morphologically the VLPs were distinct. This could be due to differences in the sequences and conformation of the envelop protein of the two different genotypes. Also it is possible that the amount of E2 protein incorporated in virus like particle could be relatively more in case of genotype 1b.

The HCV-LP genotype 3a showed almost 80% binding to Huh 7 cells, whereas genotype 1b HCV-LP showed approximately 70% binding suggesting differential affinity of the HCV-LPs towards liver cells.

The binding of HCV-LP to the Huh7 cells was maximum at 4h of incubation and after which there was decrease in fluorescence. It is possible that after 4h of incubation, the HCV-LPs enter into the cells by receptor mediated endocytosis. Interestingly, both genotype 3a and genotype 1b HCV-LPs showed similar results.

There is a cascade of events which enable the attachment and entry of HCV into permissive cells. The mAbs E8G9 and D2H3 are probably against the HCV-LP envelope protein region involved in binding to any one of the several set of cellular receptor proteins. Since the epitope for the E8G9 was putatively mapped to 596–646 which is probably structurally close to the sites of the E2 protein critical for CD81 receptor binding (∼420, 527, 529, 530, 535) [33], [34] it might have been more effective in prevention of the virus binding. The same E8G9 mAb also showed better inhibition (∼66%) of virus entry in the HCV cell culture system and the mAb H1H10 showed only marginal inhibition (∼30%). Perhaps the epitope for H1H10 is mapped to a distant location from the receptor binding domains of E2 protein. Further, mAbs D2H3, G2C7 and E1B11 didn’t show significant inhibition of binding of HCV-LP to Huh 7 cells. The epitope for D2H3 has been mapped in the region 4 (596–699 aa of E2 protein), which might be far from receptor binding sites. The epitopes for H1H10, G2C7 and E1B11 could not be mapped by western blot analysis, possibly due to the fact that the mAbs are conformation specific.

Since IgG from culture supernatant of hybridoma cells were used for the ELISA assay, it is possible that the E8G9 and H1H10 specific IgG concentration is low which is reflected in the low titrers.

Though mAb E8G9 inhibited the binding of the VLPs to Huh7 cells, the inhibition seen is not more than ∼66%. This can be attributed to the fact that HCV binding to cells involves more than one receptor. Inhibition of binding to at least the CD81 and SRB1 would be required for complete inhibition. Moreover the HCV-LPs were generated in baculovirus system; therefore the glycosylation of the insect cell expressed envelope proteins, which were earlier shown to be important for the virus entry [34], may be different when compared to HCV replicating in mammalian cells.

Earlier Keck *et al* have demonstrated the involvement of the N-terminus of HCV envelope protein E1 in virus binding and entry using a monoclonal antibody derived from this region. The mAb H111 was able to bind to HCV E1 of genotypes 1a, 1b, 2b, and 3a indicating the conservation of this epitope across the genotypes. However, still the mAb H111 could achieve only upto 70% inhibition of HCV-LP binding [35]. Additionally, Triyatni *et al*. [21] has demonstrated that several mAbs derived from multiple epitops within HVR-1could strongly bind to HCV-LP, suggesting that these epitopes are also exposed on the viral surface [21], [36]. In fact, Zibert *et al* has successfully demonstrated using patient serum that blocking of viral attachment can be revered by preincubating serum with HVR1 specific proteins. However, considering the fact that the stoichiometry of the HCV-Ab complex is not clear, they have not excluded involvement of other epitopes in viral attachment [37]. Thus it appears that multiple epitopes are required for complete neutralization, to achieve more inhibition of virus entry into target cells.

Although, the JFHI virus is derived from genotype 2a, the mAb E8G9 was able to successfully inhibit the negative strand synthesis up to 70%, suggesting that the interactions between the HCV-E2 and the Huh7.5 cells could be partially conserved. Interestingly, 100 µg/ml of mAb E8G9 showed almost 80% inhibition of input positive strand at 3hour post infection suggesting effective inhibition of the virus entry.

In conclusion, this study provides the proof of concept that mAbs can be used as a strategic approach to prevent the viral entry into target cells. However for efficient inhibition, a cocktail of mAbs are needed to completely prevent HCV infection. It would be instructive to find out if antibodies present in HCV infected patients, who do not show active infection, are able to compete with the identified neutralizing mAbs E8G9 and H1H10 in the present work.

## Supporting Information

Figure S1 **Binding efficiency of HCV-LP to human hepatoma (Huh 7) cells at 37°C at different time points.** The HCV-LPs of genotype 1b and 3a were incubated at 37°C for different time and the attachment was detected by FACS with an anti-E1E2 polyclonal antibody and FITC-conjugated anti-mouse IgG.
(TIF)

Figure S2 **Binding of HCV-LPs of genotype 1b and 3a to human hepatoma (Huh 7) cells.** Huh 7 cells were incubated with HCV-LPs (corresponding to approximately 7 µg/ml of HCV-LP) and the binding was analyzed by FACS with an anti-E1E2 polyclonal antibody and FITC-conjugated anti-mouse IgG. The MFI (shown on the X-axis) of the cell population relates to the surface density of HCV-LPs bound to the cells. The red shows the binding efficiency of 1b and black depicts 3a genotype.
(TIF)

Figure S3 **Inhibition of HCV-LP binding to Huh 7 cells using a non-specific antibody F1G4.** HCV-LP of genotype 1b and 3a were incubated with 10 µg of F1G4 mAbs taken as negative control. The Y-axis depicts the percentage activity representing both the percent binding (dark grey) and the percent inhibition (light grey) of HCV-LP attachment.
(TIF)
