# Supplementary figures and images for: Monoclonal Antibodies against Hepatitis C Genotype 3a Virus Like Particle Inhibit Virus Entry in Cell Culture System
Source: PLoS One. 2013 Jan 15;8(1):e53619. doi: 10.1371/journal.pone.0053619 (PMC3546081; doi:10.1371/journal.pone.0053619)

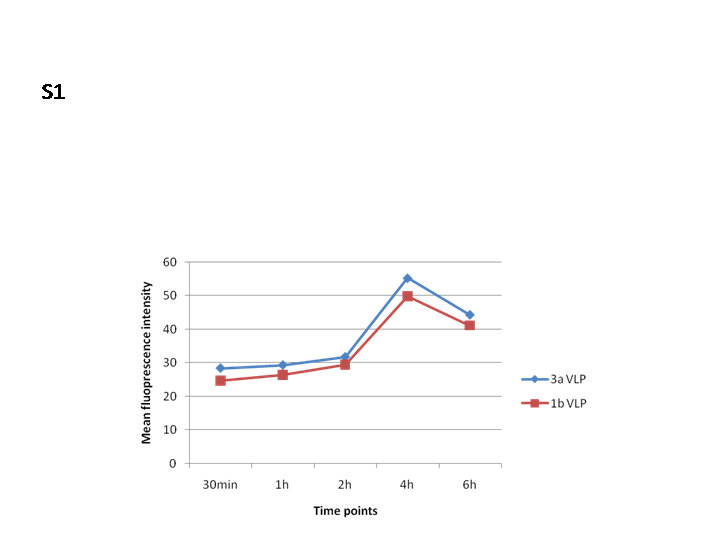

Supplement: Figure S1 — Binding efficiency of HCV-LP to human hepatoma (Huh 7) cells at 37°C at different time points. The HCV-LPs of genotype 1b and 3a were incubated at 37°C for different time and the attachment was detected by FACS with an anti-E1E2 polyclonal antibody and FITC-conjugated anti-mouse IgG. (TIF) [file pone.0053619.s001.tif]

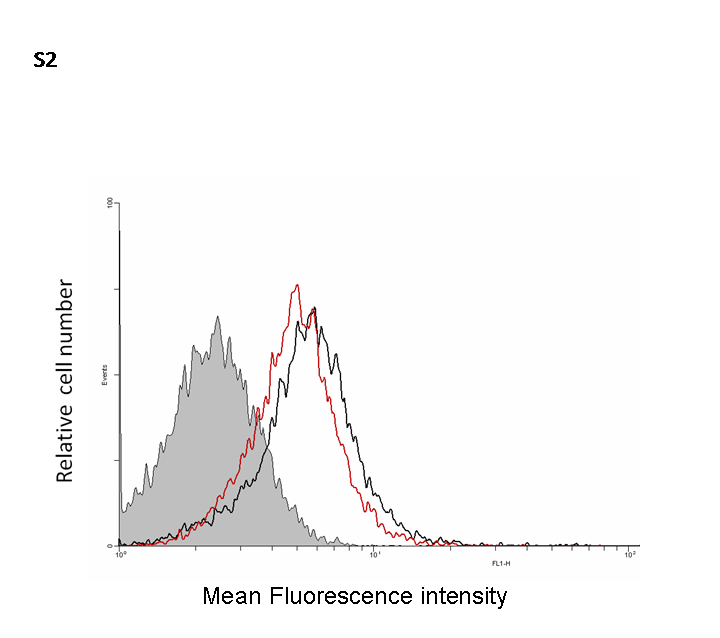

Supplement: Figure S2 — Binding of HCV-LPs of genotype 1b and 3a to human hepatoma (Huh 7) cells. Huh 7 cells were incubated with HCV-LPs (corresponding to approximately 7 µg/ml of HCV-LP) and the binding was analyzed by FACS with an anti-E1E2 polyclonal antibody and FITC-conjugated anti-mouse IgG. The MFI (shown on the X-axis) of the cell population relates to the surface density of HCV-LPs bound to the cells. The red shows the binding efficiency of 1b and black depicts 3a genotype. (TIF) [file pone.0053619.s002.tif]
